# Categorization of Third-Party Apps in Electronic Health Record App Marketplaces: Systematic Search and Analysis

**DOI:** 10.2196/16980

**Published:** 2020-05-29

**Authors:** Jordon Ritchie, Brandon Welch

**Affiliations:** 1 Medical University of South Carolina Charleston, SC United States

**Keywords:** electronic health records, medical informatics, software, interoperability, apps, app marketplace

## Abstract

**Background:**

Third-party electronic health record (EHR) apps allow health care organizations to extend the capabilities and features of their EHR system. Given the widespread utilization of EHRs and the emergence of third-party apps in EHR marketplaces, it has become necessary to conduct a systematic review and analysis of apps in EHR app marketplaces.

**Objective:**

The goal of this review is to organize, categorize, and characterize the availability of third-party apps in EHR marketplaces.

**Methods:**

Two informaticists (authors JR and BW) used grounded theory principles to review and categorize EHR apps listed in top EHR vendors’ public-facing marketplaces.

**Results:**

We categorized a total of 471 EHR apps into a taxonomy consisting of 3 primary categories, 15 secondary categories, and 55 tertiary categories. The three primary categories were administrative (n=203, 43.1%), provider support (n=159, 33.8%), and patient care (n=109, 23.1%). Within administrative apps, we split the apps into four secondary categories: front office (n=77, 37.9%), financial (n=53, 26.1%), office administration (n=49, 24.1%), and office device integration (n=17, 8.4%). Within the provider support primary classification, we split the apps into eight secondary categories: documentation (n=34, 21.3%), records management (n=27, 17.0%), care coordination (n=23, 14.4%), population health (n=18, 11.3%), EHR efficiency (n=16, 10.1%), ordering and prescribing (n=15, 9.4%), medical device integration (n=13, 8.2%), and specialty EHR (n=12, 7.5%). Within the patient care primary classification, we split the apps into three secondary categories: patient engagement (n=50, 45.9%), clinical decision support (n=40, 36.7%), and remote care (n=18, 16.5%). Total app counts varied substantially across EHR vendors. Overall, the distribution of apps across primary categories were relatively similar, with a few exceptions.

**Conclusions:**

We characterized and organized a diverse and rich set of third-party EHR apps. This work provides an important reference for developers, researchers, and EHR customers to more easily search, review, and compare apps in EHR app marketplaces.

## Introduction

The electronic health record (EHR) stores patient health information, automates clinical workflows, and supports other care-related functions such as clinical decision support [1]. Clinical and governmental drivers have facilitated widespread adoption of EHRs in health care worldwide [2]. Health care providers rely on EHRs to perform essential functions such as documenting patient encounters, providing clinical decision support, and engaging patients in their own care [3,4]. However, EHR implementation hurdles, usability flaws, and poor interoperability, among other issues, keep EHRs from delivering their full potential benefit to health care organizations [5]. The variation in EHR implementation across health organizations contributes to these problems as each organization may rely on different methods to integrate additional value into their EHR systems. Some organizations may leverage custom integration with third-party applications whereas others may resort to in-house development or other integration strategies to support their needs [6,7]. In any case these integrations tend to be time-consuming, expensive, and limited for use only within their respective organizations [8]. Ideally, a successful information technology (IT) application that integrates with an EHR at one organization would be available for the same integration with an EHR at another organization, regardless of EHR vendor [9].

The EHR app model, inspired by smartphone app marketplaces, has been proposed to increase flexibility and availability of EHR integrations while also fostering innovation in health IT [9-11]. This approach is made possible by increased EHR interoperability and standardized access to EHR data through application programming interfaces (APIs) and standards such as FHIR (Fast Healthcare Interoperability Resources) [12]. One early implementation, the SMART (Substitutable Medical Applications, Reusable Technologies) App Gallery [13] is an example of an EHR app platform that heavily leverages the FHIR standard to enable a plug-and-play style of integration with participating EHRs [14]. SMART app development depends heavily on two major concepts—apps must be both substitutable and reusable. A substitutable app accesses EHR data and can be easily added, replaced, or deleted within an EHR. This allows health care organizations to choose the app that best fits their needs [15]. A reusable app is developed once but can be installed by many clients potentially across multiple EHRs [15]. The FHIR standard serves as the common data specification that both EHR vendor APIs and SMART APIs adhere to in order to interoperate. An EHR app platform built on these concepts increases access to health IT solutions for health care organizations and allows third-party app developers to compete in a market driven by the value and price of their app [9]. Motivated by these benefits [16], major EHRs have started to create their own app marketplaces to encourage development of apps on their own platforms [15]. There are now hundreds of apps available on EHR app marketplaces.

Given the widespread utilization of EHRs and the emergence of EHR app marketplaces, it has become necessary to conduct a systematic search and analysis of apps in EHR app marketplaces in an effort to help organize, categorize, and characterize available EHR apps. This study will help health professionals, researchers, and developers understand the currently available technologies; make it easier to review, search, and compare available EHR apps; provide a common vocabulary to facilitate communication; identify where gaps and opportunities exist for research and development; and justify investment into the research and development of new EHR apps.

## Methods

### App Extraction

We identified and reviewed all known apps in public-facing marketplaces of the top 10 EHR vendors in the United States, which include (in order of market share) Epic Systems Corporation; Allscripts; eClinicalWorks, LLC; NextGen Healthcare; GE Healthcare; athenahealth, Inc; Cerner Corporation; Greenway Health LLC; Practice Fusion; and eMDs [17]. GE Healthcare did not have a public facing app marketplace at the time of this writing and Practice Fusion was recently acquired by Allscripts [18]. The leading vendors in the US market were chosen for analysis because they had publicly available app marketplaces (with the exception of GE Healthcare), and they represented a cluster of vendors serving the majority of a common set of customers.

We used custom web scrapers and public ReST (Representational State Transfer) endpoints from these EHR marketplaces to gather EHR app data such as name, description, links, website, EHR-defined app categories, ratings, reviews, EHR versions, and other available information. This information was recorded in our EHR app database wherein we consolidated duplicate EHR apps that were listed in multiple EHR marketplaces. The last data extraction occurred in February 2019. Clear indication of FHIR compatibility was not consistently present in the extracted data within or across EHR marketplaces. Marketplace offerings that offered professional services without clear evidence of EHR integration (eg, website builders or marketing services), teams of professionals granted access to EHR interfaces (eg, offsite medical coders), and medical devices without EHR integration (eg, stand-alone weight scale) were not considered apps and were excluded.

### App Review

Two informaticists (authors JR and BW) used grounded theory principles to create categories that emerged from the EHR app information [19]. EHR app classifications were created inductively by each reviewer independently based upon available information about each EHR app. Importantly, not all data fields were available across all EHRs. For example, not all marketplaces included information indicating whether the app was open source or a commercial offering. Even though some marketplaces included this information, apps were generally classified based on the information available that was common across apps from all marketplaces. When the EHR app information was either inadequate or missing, making it difficult to accurately classify the app, we referenced the app developer’s website. As common EHR app features, functions, or purposes emerged, we created categories to group similar apps. Where similarity between categories existed, they were related to form larger, more inclusive categories. Conversely, if sufficient EHR app divergence existed within a category, we subclassified the apps into more unified categories [20]. We drew category names from app descriptions, EHR-designated classifications, and common industry concepts. A minimum of three apps were required to form a category.

Following the review and initial classification by each reviewer independently, a joint review process commenced between the two reviewers. The reviewers worked together to come to a consensus on category names, the organization of the taxonomic class hierarchy, and the correct classification of each EHR app. To facilitate the joint review process, a database with all EHR app information as well as notes from each reviewer and their initial classifications were utilized. Each EHR app was reviewed and discussed together. As consensus categories emerged, formal definitions for each category were created and refined. A final classification was used to denote the consensus classification. Consensus was reached when both reviewers agreed with the classification. In cases where the reviewers failed to reach consensus, a third-party arbitrator was available. Through multiple rounds of discussion and debate, a consensus EHR app taxonomy emerged.

## Results

### App Extraction

Of the eight EHRs with public-facing marketplaces, we identified a total of 749 offerings. The total number of offerings for marketplaces ranged from 21 (eMD) to 227 (Athenahealth). In total, 153 offerings were listed on at least two EHR marketplaces, resulting in a total of 596 unique offerings; 125 were excluded from consideration for not meeting our inclusion criteria, which resulted in 471 unique apps being incorporated into our taxonomy. We categorized the EHR app into a taxonomy consisting of 3 primary categories, 15 secondary categories, and 55 tertiary categories. The three primary categories were administrative (n=203 apps, 43.1%), provider support (n=159, 33.8%), and patient care (n=109, 23.1%).

For each EHR, the distribution of apps across the primary categories followed a similar trend. In general, administrative apps make up the greatest portion of EHR apps, followed by provider support apps and then primary care apps. Interestingly, Cerner’s marketplace has a higher ratio of patient care apps, followed by provider support and then administrative. There was also a large variability in the number of listings excluded for not meeting criteria for being an app between EHR marketplaces, with eClinicalWorks accounting for 65 of 125 (52%) excluded offerings (Table 1). Each primary category is described in further detail below.

**Table 1:** Distribution of app marketplace offerings across primary categories by electronic health record (EHR) vendors.

EHR vendor	Primary category			
	Administrative, n (%)	Provider support, n (%)	Patient care, n (%)	Not an app, n (%)
Athenahealth (n=222)	90 (40.5)	63 (28.4)	45 (20.3)	24 (10.8)
eClinicalWorks (n=116)	23 (19.8)	14 (12.1)	14 (12.1)	65 (56.0)
Epic (n=113)	46 (40.7)	33 (29.2)	30 (26.6)	4 (3.5)
Allscripts (n=110)	33 (30.0)	47 (42.7)	28 (25.5)	2 (1.8)
Greenway (n=87)	48 (55.2)	19 (21.8)	4 (4.6)	16 (18.4)
Nextgen (n=37)	10 (27.0)	14 (37.8)	4 (10.8)	9 (24.3)
Cerner (n=28)	1 (3.6)	10 (35.7)	17 (60.7)	0 (0.0)
eMD (n=21)	9 (42.9)	3 (14.3)	0 (0.0)	9 (42.9)

### App Review

#### Administrative

The 203 administrative apps facilitate the administrative functions of a hospital or clinic. Within this classification, we split the apps into four secondary categories: front office (n=77, 37.9%), financial (n=53, 26.1%), office administration (n=49, 24.1%), and office device integration (n=17, 8.4%). The administrative app categories, descriptions, and counts are listed in Table 2.

**Table 2:** Category definitions and counts for administrative apps (n=203).

Categories				Category descriptions		Count, n (%)
**Administrative**				**Facilitates the conduct of administrative functions of a hospital or clinic setting**		**203 (100.0)**
	**Front office**			**Helps support front office staff interaction with patients**		**77 (37.9)**
		Scheduling	Helps schedule and manage patient appointments		32 (15.7)	
		Patient check-in	Helps manage the patient check-in process		12 (5.9)	
		Patient communication	Facilitates communication with patient for administrative purposes		12 (5.9)	
		Document management	Helps capture and manage documents		10 (4.9)	
		Answering service	Captures information related to after-hours patient calls		4 (2.0)	
		Phone triage	Facilitates triage according to industry standard protocols		3 (1.5)	
	**Financial**			**Helps manage the financial needs of the clinic**		**53 (26.1)**
		Patient billing	Captures and processes payment information from patients		20 (9.9)	
		Insurance	Facilitates claims and authorization		13 (6.4)	
		Collections	Manages patient collections		8 (3.9)	
		Medical coding	Improves accuracy and efficiency of medical coding		7 (3.4)	
		Patient pay estimation	Estimates cost of care		3 (1.5)	
	**Office administration**			**Supports the administrative needs of the clinic**		**49 (24.1)**
		Analytics and reporting	Helps track, analyze, and report on clinical operations		17 (8.4)	
		Patient experience	Measures the clinical experience of the patient		13 (6.4)	
		Inventory management	Tracks inventory of medical products		7 (3.4)	
		IT^a^ systems management	Supports the IT system needs of a health care organization		7 (3.4)	
		Compliance	Helps maintain, track, and/or report compliance		4 (2.0)	
	**Office device integration**			**Device used by office staff for administrative purposes**		**17 (8.4)**
		Scanner integration	Integrates scanners with the EHR^b^		7 (3.4)	
		Printer integration	Integrates printers with the EHR		3 (1.5)	
		Signature pad integration	Integrates a signature pad with the EHR		3 (1.5)	

^a^IT: information technology.

^b^EHR: electronic health record.

#### Provider Support

We identified 159 provider support apps, which we defined as apps that primarily support the functions of care providers in their delivery of health care to patients. Within the provider support primary classification, we split the apps into eight secondary categories: documentation (n=34, 21.3%), records management (n=27, 17.0%), care coordination (n=23, 14.4%), population health (n=18, 11.3%), EHR efficiency (n=16, 10.1%), ordering and prescribing (n=15, 9.4%), medical device integration (n=13, 8.2%) and specialty EHR (n=12, 7.5%). The provider support app categories, descriptions, and counts are in Table 3.

**Table 3:** Category definitions and counts for provider support apps (n=159).

Categories			Category descriptions	Count, n (%)
**Provider support**			**Supports the functions of care providers in their delivery of health care**	**159 (100.0)**
	**Documentation**		**Facilitates the collection and management of patient information**	**34 (21.3)**
		Dictation and transcription	Transcribes dictated clinical narratives into clinical notes	12 (7.5)
		Structured documentation	Facilitates efficient and accurate documentation	7 (4.4)
		Image capture	Captures images for documentation (usually a mobile device)	6 (3.8)
		Natural language processing	Uses natural language processing to process unstructured data	6 (3.8)
	**Records management**		**Supports access to or management of records**	**27 (17.0)**
		Image management	Allows access to or management of images, including RIS/PACS^a^ systems	11 (6.9)
		Legacy/migration	Provides access to legacy medical records or facilitates the conversion of paper records to electronic records	8 (5.0)
		Access	Consolidates patient records in one view or allows access via mobile device	5 (3.1)
		Backup	Saves data in an alternate form that can be accessed in the event of an outage	3 (1.9)
	**Care coordination**		**Helps care team members coordinate their care for a patient**	**23 (14.4)**
		Clinic scheduling	Manages the scheduling and workflow of providers in a clinic	7 (4.4)
		Service directory	Provides a list of services or providers to refer or access	7 (4.4)
		Provider communication	Facilitates the communication between care team members about a patient	6 (3.8)
	**Population health**		**Helps manage that health of a population or group of patients**	**18 (11.3)**
		Chronic care management	Helps providers manage chronic conditions in patients	10 (6.3)
		Annual wellness visit	Facilitates annual wellness visit scheduling and reporting	4 (2.5)
		Population risk assessment	Helps identify and manage at-risk patients	4 (2.5)
	**EHR^b^ efficiency**		**Makes the EHR easier or more efficient for the provider to use**	**16 (10.1)**
		Information display	Consolidates patient record into easily consumed dashboards, reports, and infographics	9 (5.7)
	**Ordering and prescribing**		**Facilitates the ordering or prescribing of a device, substance, or service**	**15 (9.4)**
		Prescription drug monitoring program	Provide access to state Prescription Drug Monitoring Program databases	5 (3.1)
		Pharmacy	Manages electronic prescription renewal and ordering	4 (2.5)
		Medical equipment	Manages electronic ordering of DME^c^	3 (1.9)
		Image ordering	Facilitates image ordering or helps manage image ordering workflow	3 (1.9)
	**Medical device integration**		**Device used by health care provider for clinical purposes**	**13 (8.2)**
		Cardiac devices	Collects data from cardiac devices	5 (3.1)
		Digital scales	Collects data from digital scales	3 (1.9)
	**Specialty EHR**		**Extends the functions of an EHR to support a specific clinical domain or specialty**	**12 (7.5)**
		Obstetrics	Extends the EHR to provide functionality for prenatal and perinatal data management	3 (1.9)
		Anesthesia	Extends the EHR to provide functionality for Anesthesia data management	3 (1.9)

^a^RIS/PACS: Radiology Information System/Picture Archiving and Communication System.

^b^EHR: electronic health record.

^c^DME: durable medical equipment.

#### Patient Care

The 109 patient care apps we identified facilitate the provision of clinical care between a health care provider and a patient. Within the patient care primary classification, we split the apps into three secondary categories: patient engagement (n=50, 45.9%), clinical decision support (n=40, 36.7%), and remote care (n=18, 16.5%). The patient care app categories, descriptions, and counts are listed in Table 4.

**Table 4:** Category definitions and counts for patient care apps (n=109).

Categories			Category descriptions		Count, n (%)	
**Patient care**			**Facilitates the provision of clinical care between a health care provider and a patient**		**109 (100.0)**	
	**Patient engagement**			**Engages patients in their own health care**		**50 (45.9)**
		Patient assessment		Collects patient-reported information for a clinical purposes		15 (13.8)
		Care plan management		Helps patient follow a provider's rehab instructions, medication schedule, and/or care plan regimen		10 (9.2)
		Patient education		Provides education and instruction resources to patients specific to their care		9 (8.3)
		Health record access		Allows patients to access, download, and share their medical records, or allows providers to fulfill medical record requests		6 (5.5)
		Patient wearables		Records information from patient wearables (passive involvement)		6 (5.5)
	**Clinical decision support**			**Provides or delivers decision support to providers based on patient data**		**40 (36.7)**
		Ordering CDS^a^		Supports the appropriateness of medication, imaging, and lab test orders		10 (9.2)
		Medication CDS		Provides decision support for medication dosing and monitoring		9 (8.3)
		Patient risk assessment		Assess a patient's health risk		5 (4.6)
		Knowledge management		Provides access to and manages medical knowledge for providers		5 (4.6)
		Patient monitoring		Monitors health of patient and alerts provider of notable changes		4 (3.7)
	**Remote care**			**Supports the provision of care to patient remotely**		**18 (16.5)**
		Telehealth platform		Gives a provider technical capabilities to meet with a patient remotely		12 (11.0)
		Remote consult		Provides access to care providers or specialists who are remote		5 (4.6)

^a^CDS: clinical decision support.

## Discussion

### Principal Results

We conducted a systematic search and analysis of apps in EHR app marketplaces to help organize, categorize, and characterize available EHR apps. This study brings value to the health IT industry because it creates a common vocabulary that can be used to communicate about EHR apps, helps health care organizations identify EHR app solutions, and justifies investment into the research and development of new EHR apps. With this study, we can identify common patterns of EHR integration approaches and create a template to streamline future EHR app development and integration. This helps researchers identify gaps in integration capabilities, standards, or functionalities that need to be addressed by governing bodies, standards organizations, EHR vendors, or EHR app developers.

Our EHR app review organized and characterized 471 unique EHR apps into 3 primary categories, 15 secondary categories, and 55 tertiary categories. Several categories were larger or more well-defined than others. Administrative apps represented the largest share of EHR apps with 203 apps. Provider support and patient care apps were the other primary categories with 159 and 109 EHR apps, respectively. EHR marketplaces tended to reflect this overall trend with the majority of apps falling under the administrative category, followed by provider support and patient care. Cerner followed a distinctly different trend with patient care representing the majority of their apps and only a single administrative app. This may in part be attributed to how Cerner validates apps that are submitted by third-party developers. While all app galleries reviewed here have a submission process, and several list disclaimers that not all submissions may be listed upon submission, Cerner has an additional validation step. Apps that don’t meet a certain standard set by Cerner may be rejected or asked to resubmit after outstanding issues are resolved. This adds extra rigor in the Cerner app submission process that may account for the lower total app count in their gallery as well as the different ratio of primary categories observed. The fact that zero offerings in Cerner’s gallery were considered “Not an app” and excluded from consideration in our search and analysis may be attributed to their unique validation approach and, generally speaking, Cerner’s apps required less attention when assigning apps to categories. However, Cerner also had fewer total offerings listed in their gallery (n=28) than all other vendors except for eMD (n=21). App quality is an important issue and while the right amount of validation is difficult to quantify [8], it is important to note that if validation is too strict, it could suppress innovation and defeat a key purpose of the app model, which allows competition among developers based on app value and price [9]. This allows clients to validate app offerings and reward innovation and the value the apps provide [15].

Interestingly, provider support apps had the greatest variability and ambiguity among the three primary categories of apps. Provider support had more secondary categories than the other two primary categories combined, accounting for 8 of the 15 (53%) total secondary categories. Many apps offered multiple functionalities or had feature sets that made it difficult to assign secondary and tertiary categories. The value these apps provided and how they were intended to be used by the provider was often unclear. Provider support accounted for 63% (14/22) of apps that were not specific enough to place in a secondary category. Additionally, provider support accounted for 46% (25/54) of apps that were not specific enough to place in a tertiary category. The secondary categories under provider support with the most apps without tertiary categories were specialty EHR (6/12, 50%), EHR efficiency (7/16, 44%), and medical device integration (7/13, 39%). Among all other secondary categories, office device integration had the highest percentage of apps that did not fall into a tertiary category (4/17, 24%), followed by clinical decision support (7/40, 18%). This suggests that the provider support category has the greatest need for further refinement and innovation out of the three primary categories.

The app model is a remedy to the one-size-fits-all strategy that is failing to meet the needs of patients, providers, and administrators in a rapidly evolving landscape [8]. For the app model to be effective, the apps listed need to solve a clearly defined problem instead of offering diverse sets of features and functionalities that begin to approximate a one-size-fits-all-solution. In other words, it needs to be obvious what category the app belongs in. The taxonomy of apps we have curated will help health IT companies and app developers match app development to a well-defined purpose and assist health professionals in identifying gaps in the current set of app categories. As app functionality and feature sets become more cohesive and achieve alignment with specific EHR app categories, the value and impact the app model will have on health care will increase as patients, providers, and administrators can more easily search, install, and ultimately be the market force that will drive innovation and value of EHR apps.

### Limitations

We acknowledge several shortcomings of the current review. First, our review is limited only to apps currently available on the top 10 EHR app marketplaces by market share in the United States. We acknowledge there are other EHR vendors worldwide developing app marketplaces as well that were not reviewed here. During our review and research of EHR apps, we came across several other apps that claimed EHR integration that would have been included if they had been listed in an EHR marketplace. It would be unfeasible to know all apps that integrate with EHRs; nevertheless, we hope that as EHR marketplaces mature, these apps will become listed in the EHR marketplaces and organized in our review. Second, several apps had characteristics or features that could justify their classification under more than one category. In these cases, we endeavored to classify apps to the lowest level in the taxonomy as possible while still accurately reflecting the apps’ primary purpose, which sometimes resulted in higher level classifications. In a few instances, when the information was insufficient to determine whether an offering was an app, we erred on the side of inclusion. As a result, a few apps in our taxonomy may have been inappropriately included. As further information becomes available, their inclusion and classification will be re-evaluated. Third, the review was conducted by two informaticists. We recognize that shortcomings, inaccuracies, and/or bias may exist in the interpretation and characterization of the apps. Independent input from a panel of expert stakeholders would increase robustness and validity of the EHR app review. Finally, the EHR app review represents a single point in time (February 2019). However, as new EHR apps get added to marketplaces and new app information becomes available, the results will become outdated. We anticipate conducting this review again in a few years to understand how EHR app marketplaces have evolved over time.

### Comparison With Prior Work

The SMART app model was proposed in 2009 by Mandl et al [9]. Since then, EHR vendors have followed suit by building their own app marketplaces. We identified hundreds of apps in these marketplaces that allow integration with their respective EHR vendor. Current EHR marketplaces do not fully reflect the original proposal made by Mandl et al [9], which called for total substitutability of apps across any EHR by conforming to a single standard. Without conforming to a single standard as proposed by Mandl et al [9], each app would need to integrate with each EHR marketplace individually, as is the case today. For instance, we observed that 153 apps integrate with two or more EHRs. This does not quite meet the proposal made by Mandl et al [9] where an app lives on a single platform and can be integrated with any health system regardless of EHR vendor.

### Conclusions

We characterized and organized a diverse and rich set of third-party EHR apps. This work provides an important reference for developers, researchers, and EHR customers to more easily search, review, and compare apps in EHR app marketplaces. While future research and validation among independent informaticists and stakeholders will increase the validity and value of this review, this work provides a strong foundation upon which future EHR app research will be established.

## Abbreviations

API: application programming interface
CDS: clinical decision support
DME: durable medical equipment
EHR: electronic medical record
FHIR: Fast Healthcare Interoperability Resources
IT: information technology
ReST: Representational State Transfer
RIS/PACS: Radiology Information System/Picture Archiving and Communication System
SMART: Substitutable Medical Applications, Reusable Technologies
